# Biogenic-Synthesized Silver Nanoparticles Using the *Ligilactobacillus salivarius* KC27L Postbiotic: Antimicrobial, Anti-Biofilm, and Antioxidant Activity and Cytotoxic Effects

**DOI:** 10.1007/s12602-025-10481-x

**Published:** 2025-02-26

**Authors:** Zehranur Yuksekdag, Reyhan Kilickaya, Filiz Kara, Berat Cinar Acar

**Affiliations:** 1 Faculty of Science, Department of Biology, Gazi University, Ankara, Turkey; 2 Faculty of Engineering, Department of Industrial Engineering, Baskent University, Ankara, Turkey

**Keywords:** Microbial synthesis, *Ligilactobacillus salivarius*, Postbiotic, Silver nanoparticles, Biological activity, Cytotoxicity

## Abstract

This study aimed to synthesize silver nanoparticles (AgNPs) using the postbiotic of the *Ligilactobacillus salivarius* KC27L strain and evaluate their multifunctional biological properties. The use of *L. salivarius*, a probiotic bacterium known for its ability to produce a wide range of metabolites, plays a crucial role in this process by acting as a natural, eco-friendly reducing, and stabilizing agent during AgNP synthesis. This approach not only eliminates the need for hazardous chemicals typically used in nanoparticle synthesis but also enhances the biocompatibility and biological efficacy of the resulting nanoparticles. Synthesized AgNPs were analyzed by Fourier transform infrared spectroscopy, FTIR (metabolites of postbiotic); UV–vis (peak of 435 nm); scanning electron microscope, SEM; transmission electron microscopy, TEM (spherical shapes, sizes < 50 nm), energy-dispersive spectrometry, EDS (peak at 3 keV); and zeta potential (− 18.6 mV). These nanoparticles (0.156–40 mg/mL) were evaluated for the antimicrobial and anti-biofilm activities against *Escherichia coli* ATCC 11229, *Pseudomonas aeruginosa* ATCC 27853, *Staphylococcus aureus* ATCC 25923, *Staphylococcus epidermidis* ATCC 35984, and *Streptococcus mutans* ATCC 25175, and antioxidant activities using four different methods (2,2-diphenyl-1-picrylhydrazyl free radical scavenging, metal ion chelating, hydroxyl radical scavenging, and superoxide anion scavenging activities). Also, the cytotoxic activity was investigated against a normal cell line (L929) for 24, 48, and 72 h. At a concentration of 40 mg/mL, the AgNPs demonstrated the highest antimicrobial efficacy, with inhibition zones measured as 14.9 mm for *P. aeruginosa*, 9.5 mm for *E. coli*, 15.7 mm for *S. epidermidis*, and 12.9 mm for *S. mutans*. The AgNPs exhibited anti-biofilm activities against all Gram-positive and Gram-negative bacteria strains studied. According to the DPPH method, the highest antioxidant activity was determined at 40 mg/mL AgNP concentration (80.93%). AgNPs were found to have no toxic effect at low concentrations (0.39–25 µg/mL). Biogenic synthesized AgNPs could be used in biotechnological applications (biomaterials, health, environmental, etc.) with antibacterial, anti-biofilm, antioxidant, and nontoxic properties. However, further research is needed to understand the mechanisms of action of the particles fully.

## Introduction

AgNPs have attracted great interest due to their unique properties and have evolved into a wide range of applications in various sectors such as cosmetics, textiles, food processing and packaging, medical devices, diagnostics, and therapeutics (such as drug release, wound dressings, and development of antibacterial and anticancer agents) [1].

Various physical and chemical methods are widely used for the synthesis of AgNPs. However, stability and the use of toxic chemicals are major concerns. The use of toxic chemicals on the surface of metal nanoparticles and non-polar solvents in the synthesis procedure limit their application areas [2]. Although traditional physical and chemical methods, often expensive and involving toxic chemicals harmful to the environment and human health, have long dominated AgNPs synthesis, recent advancements have revealed the promising potential of biological methods utilizing microorganisms such as fungi, algae, bacteria and their metabolites, and plant extracts [3–5]. As the demand for silver nanoparticles increases in various applications such as medicine, electronics, and environmental management, there is a critical need to develop sustainable, green synthesis methods that minimize environmental impact. Microbial and postbiotic-based synthesis of silver nanoparticles offers a promising alternative by offering a more environmentally friendly approach that utilizes natural resources and avoids hazardous chemicals. This method aligns with the principles of green nanotechnology, which focuses on ensuring safer production processes while also minimizing environmental impact [6]. For nanoparticle production, green methods utilize biological substances as reducers (converting metal ions), capping agents and stabilizers (controlling shape, size, and preventing clumping), and ligands (coating the nanoparticle surface and passivating the metal) [7].

Microorganisms and their metabolites synthesize nanoparticles by reducing metal ions to elemental metal. Microbial synthesis is classified into intracellular and extracellular synthesis based on the location where nanoparticles are produced. The intracellular synthesis method occurs through the transport of metal ions into the cell by enzymes. In the biosynthesis mechanism, silver ions enter the microbial cell and undergo reduction through electrostatic and enzymatic interactions, leading to nanoparticle formation within the cell. Extracellular synthesis, on the other hand, occurs through the direct interaction of metabolites (enzymes and proteins) found in the culture supernatant with metals [8].

Studies have been reported to determine the biological properties of AgNPs synthesized in lactic acid bacteria, such as antimicrobial activity [9, 10], anti-biofilm activity [11, 12], antioxidant activity [13, 14], and cytotoxicity [15]. Size, shape, charge, formation, and surface modification can affect the biological activity of AgNPs. AgNPs are effective even at very low concentrations, minimizing the potential for tissue toxicity due to silver release (in particulate and/or Ag^+^ form). However, it has been revealed that the current potential toxicity of AgNPs is not only associated with metal accumulation, but also with the synthetic methods used in their synthesis [16].

Postbiotics are a diverse group of metabolites produced by microorganisms. These can include (i) soluble factors released by live or lysed bacteria, such as enzymes, peptides, and organic acids; (ii) metabolic byproducts with beneficial effects on the host; and (iii) compounds derived from food or microbial components, including whole or broken-down cells, that promote health when consumed in sufficient amounts. Postbiotic, encompassing both structural and metabolic byproducts of microorganisms, include a diverse range of molecules like teichoic acid, short-chain fatty acids, vitamins, enzymes, exopolysaccharides, peptides, amino acids, and fermentation products [17]. *Ligilactobacillus salivarius*, a Gram-positive, homofermentative bacterium previously known as *Lactobacillus salivarius*, is gaining attention as a promising probiotic due to its diverse functional properties, including antimicrobial activity, immune modulation, and the ability to influence gut microbiota [18].

This study aimed to synthesize biogenic AgNPs via postbiotic (metabolites) of the *L. salivarius* strain (KC27L). The physical properties of the obtained AgNPs were characterized using UV–visible spectroscopy, SEM, TEM, and Zeta Sizer, and the chemical properties were analyzed using FTIR and EDS. The antimicrobial and anti-biofilm activities of AgNPs against Gram-negative *Pseudomonas aeruginosa* and *Escherichia coli*, Gram-positive *Staphylococcus aureus*, *Staphylococcus epidermidis*, *Streptococcus mutans*, antioxidant activity, and in vitro cytotoxic properties in cell culture were determined.

## Materials and Methods

### Bacteria and Chemicals

The metal oxide precursor, silver nitrate (AgNO_3_), was purchased from Isolab (Germany). All bacteria used in the study were obtained from the Gazi University, Biotechnology Laboratory Collection for stocks culture collection. The microorganism, *Ligilactobacillus salivarius* KC27L, which was isolated previously from chicken feces, identified by 16S rRNA and biochemical analyses [19], was used to synthesize AgNPs. MRS (De Man, Rogosa and Sharpe) broth/agar (Oxoid, Thermo Fisher Scientific Inc, Basingstoke, UK) at 37 °C for 48 h was used to culture the strain. Gram-negative *Escherichia coli* ATCC 11229, *Pseudomonas aeruginosa* ATCC 27853 and Gram-positive *Staphylococcus aureus* ATCC 25923, *Staphylococcus epidermidis* ATCC 35984, *Streptococcus mutans* ATCC 25175 were used as test bacteria for biological activities determinations. The pathogens were sub-cultured on nutrient broth/agar (NB, Merck, Merck KGaA Darmstadt, and Germany). For the experiments, bacterial cultures were adjusted to ~ 10 log cfu/mL using the McFarland device (Biosan DEN-1) in PBS (phosphate-buffered saline, 1.44 g Na_2_HPO_4_, 0.24 g KH_2_PO_4_, pH 7.4) and evenly inoculated into the culture media.

### Preparation of Postbiotic

The *L. salivarius* KC27L culture was incubated in MRS broth at 37°C for 48 h. To remove bacterial biomass, the culture was centrifuged at 10,000 rpm (Sigma 2-16KC) for 10 min and filtered through membrane filter with a 0.2-μm pore dimension in three repetitions. The postbiotic was then collected and stored at 4°C for nanoparticle synthesis [4].

### Synthesis and Optimization

The biogenic synthesis of AgNPs using the postbiotic of *L. salivarius* KC27L was studied to obtain the optimum biosynthetic conditions by determination of five factors: reaction time (6, 12, 24, 48, and 72 h), concentration of AgNO_3_ solution (1, 3, 5, 7, and 10 mM), volume ratio of the postbiotic to AgNO_3_ solution (1:1, 1:2, 1:3, 1:4, and 1:5), pH of the postbiotic (3.7, 7, and 10), and temperature (20, 25, 30, 35, 40, and 45 °C). The aqueous solution of different concentrations of AgNO_3_ was treated with postbiotic incubated to synthesize AgNPs. During the optimization of each parameter, the values of the other parameters were held constant. For instance, to optimize the reaction time for the biogenic synthesis of AgNPs using the postbiotic of *L. salivarius* KC27L, a series of incubations were conducted at different time intervals (6, 12, 24, 48, and 72 h). In each trial, an equal volume of postbiotic was mixed with a 5 mM AgNO_3_ solution in a 1:1 ratio. The mixture was then incubated at a constant temperature of 30 °C, with the pH adjusted to 7. The optimal reaction time was determined by comparing the nanoparticle formation efficiency at each time point. The formation of AgNPs was visually identified by following color change and monitored using a UV–vis spectrophotometer (Digilab Hitachi U-1800) at different wavelengths (300–700 nm). The development of a yellow–brown color suggests the successful synthesis of AgNPs [20]. The synthesized nanoparticles were subjected to extensive washing with water to eliminate residual metal ions or other impurities. According to the UV–vis results under the conditions mentioned above, the condition giving a sharper absorbance peak was accepted as the optimum condition. The AgNPs synthesized under these conditions were further used for characterization and biological activity studies.

### Characterization

The morphology and size of AgNPs were determined by SEM coupled with EDS for element mapping (QUANTA 400F Field Emission; FEI Company, Hillsboro, Oregon 97,124, USA) operating at a 1.2-nm resolution. The microstructure of thin layers containing AgNPs was precisely characterized using a high-vacuum mode in the FEI Tecnai G2 Spirit BioTwin CTEM, with an acceleration voltage ranging from 20 to 120 kV. Zeta potential measurement is a critical analysis technique because it provides complementary information to other properties. This information is crucial for understanding, physical stability, and behavior in colloidal dispersions [20]. The Zeta sizer Nanoseries (Malvern CGS-3 instrument) measured zeta potential and hydrodynamic dimension of the synthesized nanoparticles.

FTIR spectrometer (Bruker, Vertex 70 V, and USA) was used to identify the functional groups and biomolecules responsible for the reduction of silver ions and to detect potential metabolites of the postbiotic. All spectra were taken with 32 scans at a resolution of 4 cm^−1^ which has a working range of 500–4000 cm^−1^. Elemental composition of AgNPs was determined by EDS.

### Antibacterial Activity

The antibacterial activity of AgNPs against Gram-negative *E. coli*, *P. aureginosa* and Gram-positive *S. aureus*, *S. mutans*, and *S. epidermidis* pathogens were evaluated by agar well diffusion method [21, 22]. Active pathogen cultures were adjusted to McFarland 0.5, and 100 μL was transferred onto sterile Petri dishes. Then, approximately 20 mL of Muller-Hinton (MH, Merck) solid medium was transferred to sterile Petri dishes and inoculated by the pour plate method. On the frozen solid medium, 100 μL of AgNPs at final concentrations of 0.625, 1.25, 2.5, 5, 10, 20, and 40 mg/mL were added to the wells and incubated at 37 °C for 24 h. At the end of incubation, the inhibition zone diameter was measured with calipers.

### Anti-Biofilm Activity

Biofilm production of pathogenic bacteria (*E. coli*, *P. aureginosa*, *S. aureus*, *S. mutans*, and *S. epidermidis*) used in the study was determined colorimetrically based on crystal violet staining [23]. Two hundred microliters (µL) of pathogenic bacteria, adjusted to an optical density of 0.05 at 600 nm, were dispensed into each well of a 96-well microplate. The microplate was incubated in a static condition at 37 °C for 24 h. At the end of the incubation periods, the wells were aspirated and washed three times with sterile saline (0.9% NaCl; Merck) to remove planktonic cells. The wells dried at room temperature were fixed by adding 150 μL each of 95% methanol (Merck, Germany). Two hundred microliters of 0.1% crystal violet solution (Merck, Germany) was transferred to the wells and kept at 25℃ for 30 min. The wells were rinsed with sterile distilled water to eliminate any non-adherent dye. A 33% solution of glacial acetic acid (Merck, Germany) was added to the wells, and the dye attached to the biofilm structures on the well walls was dissolved. Readings at a 570-nm wavelength determined the optical density of the crystal violet color of the dissolved dye. Biofilm production levels of pathogenic bacteria were categorized as weak (ODcut-off < OD ≤ 2 × ODcut-off), moderate (2 × ODcut-off < OD ≤ 4 × ODcut-off), or strong (OD > 4 × ODcut-off).

A stock solution of AgNPs (50 mg/mL) was prepared in dH_2_O and sonicated to achieve a homogeneous mixture. The stock was then diluted to create a range of concentrations from 0.156 to 40 mg/mL. The assay was carried out using the previously described crystal violet protocol. Wells containing culture medium without AgNPs was used as the control. The percentage of biofilm inhibition was calculated using the formula [24]:

$${\%} \;\text{Percentage inhibition }= [({\text{OD}}_{\text{control}}-{\text{OD}}_{\text{experimental}})/ {\text{OD}}_{\text{control}}] \times 100$$

### Antioxidant Activity

The antioxidant activity of different concentrations (0.156–40 mg/mL) AgNPs synthesized by biogenic synthesis was determined by 2,2-diphenyl-1-picrylhydrazyl (DPPH) free radical scavenging, metal (Fe^+2^) ion chelating, hydroxyl radical scavenging, and superoxide anion scavenging activities.

The free radical scavenging ability of AgNPs was assessed using a DPPH solution of AgNPs synthesized in 95% methanol. The stock solution was used to prepare 2-mL samples of the test solution, with concentrations ranging from 0.156 to 40 mg/mL. One milliliter (1 mL) of the DPPH reagent was added to the test samples. The mixtures were then shaken vigorously and incubated in the dark for 30 min. Ascorbic acid (AA) was used as a positive control, while methanol served as a blank. The absorbance of all samples was measured at 517 nm using a UV-vis spectrophotometer (U-1800, HITACHI). The equation below was used to calculate the percent inhibition of the DPPH radical [24]:

$$\text{Scavenging activity }({\%}) = [\text{B}-(\text{ S}-\text{Sc})]/\text{B }\times 100$$

B: blank
S: sample
Sc: control

The ability of ferrous ions to chelate was measured using ferrozine, a compound that forms a pink-red colored complex with a maximum absorbance at 562 nm [25]. AgNPs at different concentrations (0.156 to 40 mg/mL) were added to 1 mL of PBS. To this mixture, FeCl_2_ solution (2 mM, 0.05 mL) and ferrozine solution (5 mM, 0.2 mL) were added (10 min at 25℃), and the absorbance was measured. Lower absorbance indicates higher chelating capacity. The ferrous ion (Fe^2+^) chelating ability of the test extracts was compared to that of ascorbic acid. The formula given in the DPPH method was utilized to assess the metal chelation activity.

The hydroxyl radical scavenging activity was determined using a method where hydroxyl radicals, generated from the reaction between ions and hydrogen peroxide and hydroxylate salicylic acid. This reaction forms a pink-violet compound with a maximum absorbance at 624 nm [26]. Microbial-synthesized AgNPs were suspended in 1 mL of PBS. Then, 1 mL of brilliant blue solution (0.435 mM), 2 mL of FeSO_4_ solution (0.5 mM), and 1.5 mL of H_2_O_2_ solution (3% w/v) were added to the samples. The mixture was incubated at 37°C for 1 h, followed by centrifugation at 4000 rpm for 5 min. Finally, the absorbance of the samples was measured. The formula given in the DPPH method was utilized to assess the hydroxyl radical scavenging activity.

The superoxide anion scavenging activity at different concentrations (0.156 to 40 mg/mL) of AgNPS was assessed using a system of pyrogallol autoxidation under alkaline conditions [27]. The samples were mixed with 1.5 mL of 0.05 M Tris-HCl buffer (pH 8.2) in the dark at 25°C for 10 min. One hundred microliters of 30 mM pre-heated pyrogallol (25°C) was then added to the mixture. The reaction was stopped of concentrated HCl (0.5 mL) and the absorbance of the samples was measured at 329 nm. The formula given in the DPPH method was utilized to assess the superoxide anion scavenging activity.

### Cytotoxicity

Cytotoxicity of AgNPs on normal fibroblast cells (L929) was determined by a MTT (3-(4,5-dimethylthiazol-2-yl)−2,5-diphenyltetrazolium bromide) colorimetric test at different concentrations (0.39–200 µg/mL) and time (24, 48, and 72 h) [28]. The analysis was conducted using services provided by Ankara Yıldırım Beyazıt University Central Research Laboratory. Untreated cells were used as a control and loss of viability was calculated using the following equation.

$$\text{Cell viability} \;{\% }= [1 ({A}_{t}-{A}_{b})/({A}_{c}-{A}_{b})] \times 100\;{\%}$$

where *A*_*t*_ represents the absorbance of the sample, *A*_*b*_ represents the bleaching absorbance without the sample, and *A*_*c*_ represents the absorbance value of the control.

In this study, the cytotoxic effects of the applications were assessed in accordance with ISO 10993-5 standards. According to these standards, cell death of ≥50%, 21–50%, 11–20%, and ≤10% is considered as high cytotoxic effect, moderate effect, low effect, and non-cytotoxic effect, respectively. Also, the results were used to determine the IC_50_ value.

### Statistics

The studies were conducted with two parallels and two replicates, although the number of parallels varied for each study. The results obtained from the studies were presented as the mean ± standard deviation (SD) of these replicates. SPSS 22.0 software (SPSS Inc., Chicago, IL, USA) was used for statistical analyses.

## Results

### Synthesis and Optimization

**Fig. 1:**
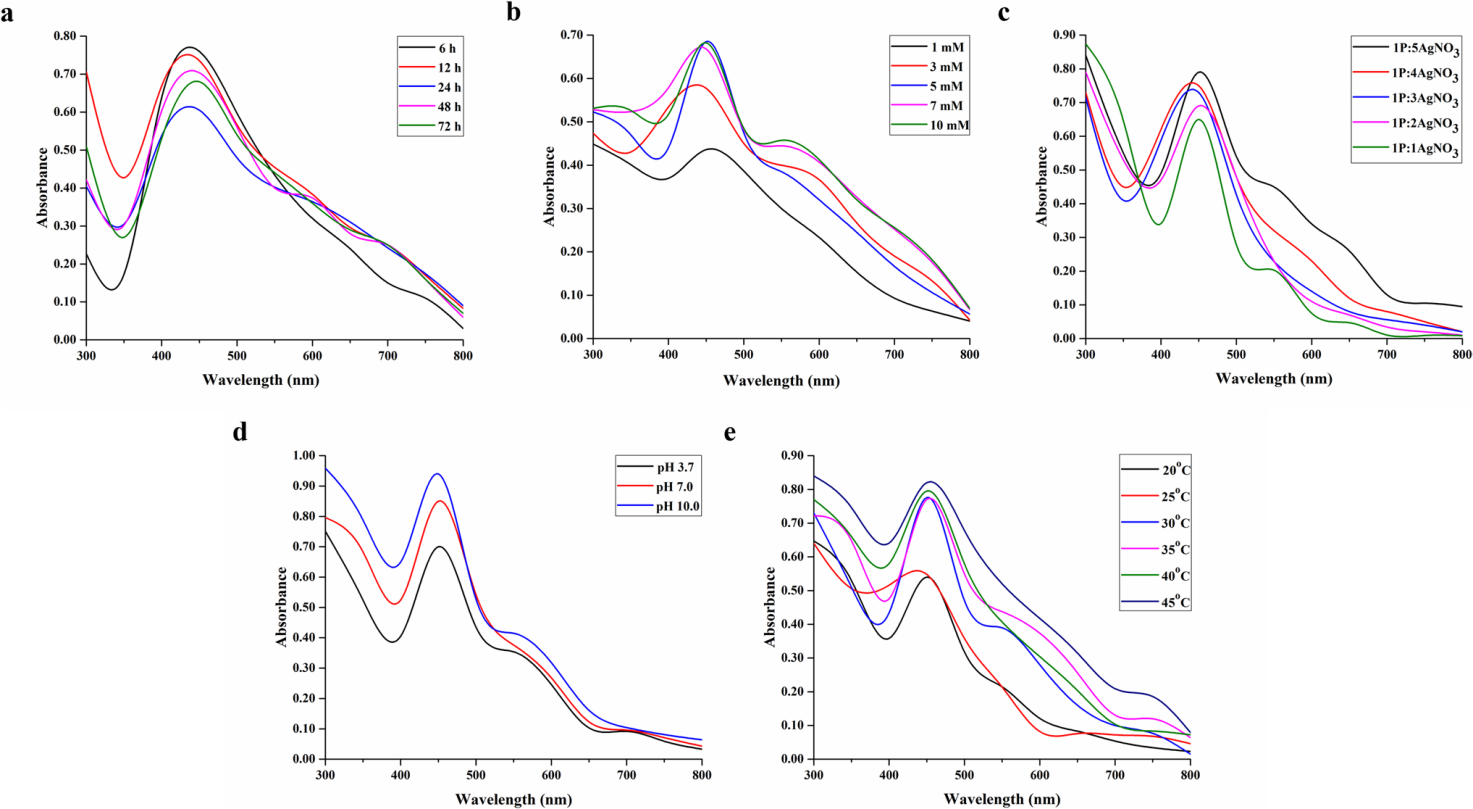
UV–visible spectra for the optimization of AgNP synthesis parameters: **a** reaction time, **b** AgNO_3_ concentrations, **c** volume ratio of the postbiotic to AgNO_3_, **d** pH, **e** temperature

The color change from yellowish brown to dark brown in the solution caused the formation of AgNPs through the reduction of the silver salt at the end of the reaction of the postbiotic with AgNO_3_. Various parameters optimized for the biosynthesis of AgNPs, such as contact time, AgNO_3_ concentration, pH, temperature, and the concentration of bacterial supernatant. Figure 1a exhibit the UV–vis spectra of AgNPs synthesize at different time intervals (6–72 h). The sixth hour, when AgNPs reached the highest density, was selected as the optimum time. The increase in the intensity of the wavelength absorbance is a result of the reduction of silver ions, increasing the number of nanoparticles. The appropriate concentration of AgNO_3_ was determined to maximize and stabilize the production of AgNPs (1, 3, 5, 7, and 10 mM). The absorption spectra (Fig. 1b) showed that 5 mM AgNO_3_ solution had the highest intensity of the wavelength absorbance and narrow peak. Altering the ratios of supernatant to AgNO_3_ from 1:1 to 1:5 (v/v) led to an increase in the intensity of the absorbance peak and enhanced the synthesis of AgNPs (Fig. 1c). Compared to the other combinations, the synthesis in the 1:1 ratio was chosen as the optimum condition as it had the narrowest peak, suggesting that the AgNPs obtained were mono-dispersed. When the effect of pH on nanoparticle synthesis was examined, it was observed that absorbance increased as pH increased (pH 3.5, 7, 10) (Fig. 1d). The peaks are similar, so the optimum pH was chosen as 3.5, the natural pH of the supernatant. When the effect of temperature on the nanoparticles was examined, peaks with wider and higher absorbance were observed as the temperature increased from 20 to 45 °C (Fig. 1e). This is an indication that the yield and polydispersity increase as the temperature increases. A temperature of 30 °C was chosen as the optimum temperature as it represents the monodisperse nanoparticle with narrower peak and the yield is also good from the absorbance value.

**Fig. 2:**
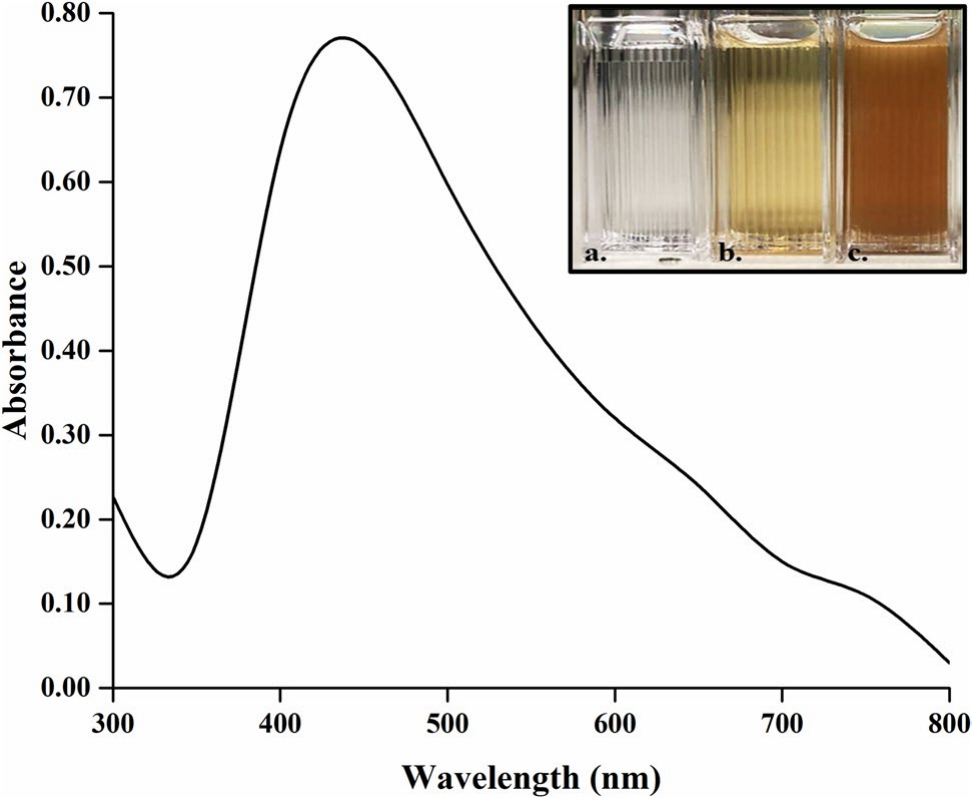
UV–visible spectrum of AgNPs synthesized at optimum conditions. (a) AgNO_3_, (b) postbiotic, and (c) AgNP

Optimum conditions for synthesis of AgNPs were determined as 1:1 ratios of supernatant to AgNO_3_, 5 mM AgNO_3_ concentration, 6-h reaction time, pH 3.5, and 30 °C temperature. AgNPs synthesized under optimum conditions were used in subsequent studies (Fig. 2).

### Characterization

**Fig. 3:**
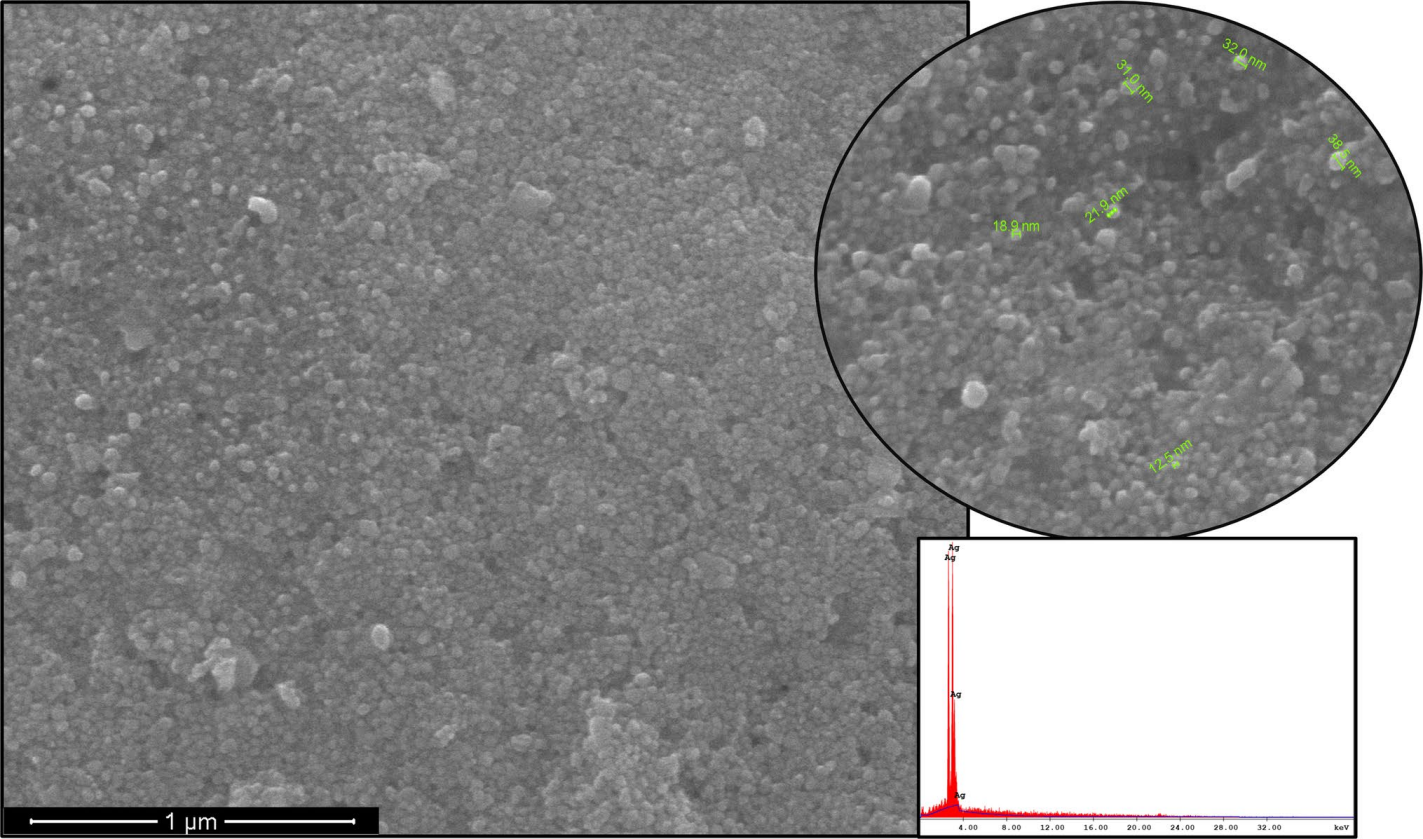
SEM micrograph and EDS spectra of AgNPs

**Fig. 4:**
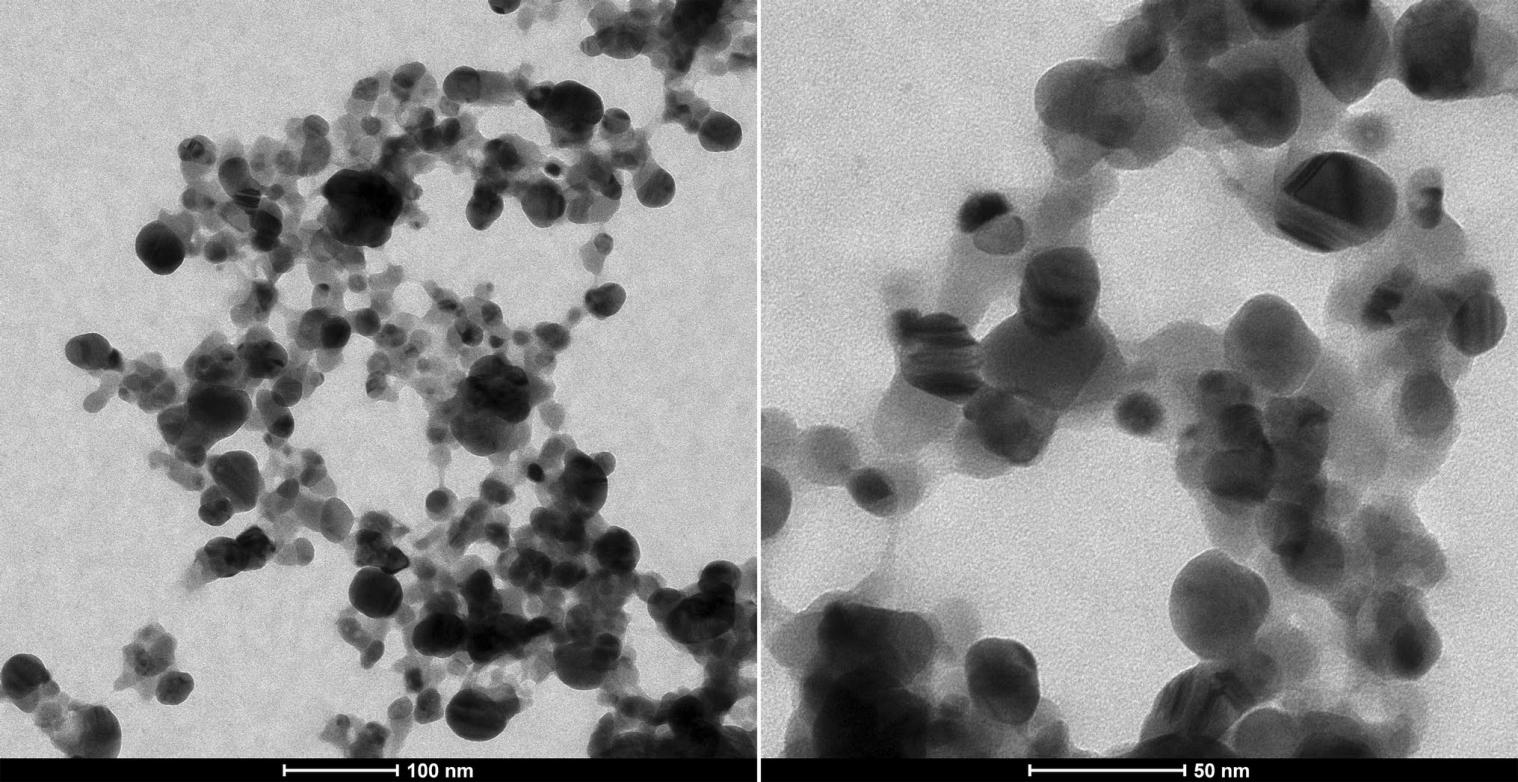
TEM micrograph of AgNPs

**Fig. 5:**
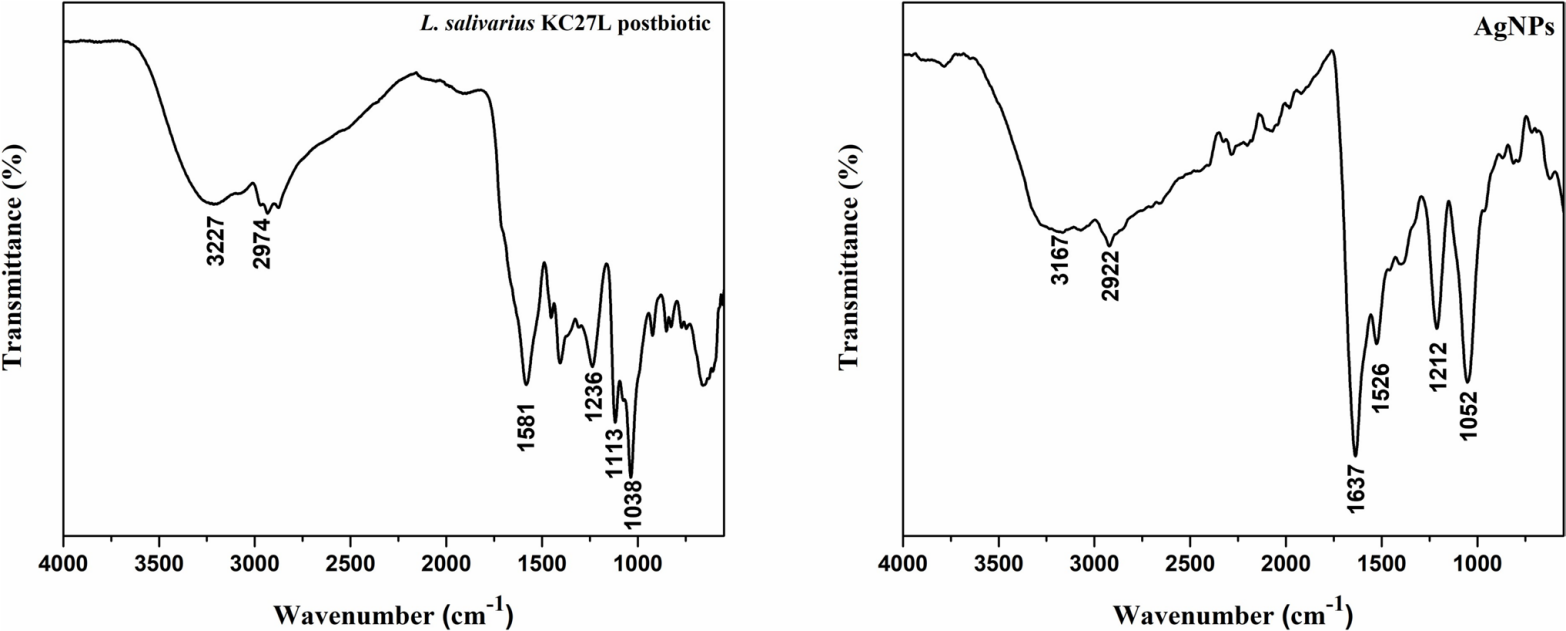
FTIR absorption spectrum of AgNPs and *L. salivarius* KC27L postbiotic

According to the SEM results (Fig. 3), AgNPs are spherical; most particles have sizes ranging from 10 to 40 nm and agglomerated. Also, TEM measurements (Fig. 4) confirm that the AgNPs have similar properties (particles sizes < 50 nm). In addition, biomolecules coat the surfaces of the nanoparticles. The average size and polydispersity index (PDI) of AgNPs were 110 nm and 0.309, respectively. The zeta potential result is − 18.6 mV with a single peak. The stretching vibrations of hydroxyl groups (− OH) and amine groups (− NH_2_) are indicated by the broadband at 3167 cm^−1^ in Fig. 5, implying their role in AgNP synthesis.

### Antibacterial activity

The antimicrobial activity of biogenic AgNPs was evaluated against the Gram-negative bacteria *E. coli* and *P. aeruginosa*, and the Gram-positive bacteria *S. aureus*, *S. mutans*, and *S. epidermidis* at different concentrations (0.625–40 mg/mL). The average diameter (in millimeters) of the inhibition zones for each AgNPs concentration, determined from three replicates, is presented in Table 1. Biogenic AgNPs at 40 mg/mL were most effective in inhibiting the growth of *S. aureus* ATCC 25923 (19.0 mm). The 0.625 mg/mL AgNPs exhibited lowest zones of inhibition against *E. coli* ATCC 11229 (7.7 mm). The inhibition zone for AgNPs at a concentration of 40 mg/mL was measured as 14.9 ± 0.2 mm for *P. aeruginosa*, 9.5 ± 0.4 mm for *E. coli*, 15.7 ± 0.1 mm for *S. epidermidis*, and 12.9 ± 0.4 mm for *S. mutans*.

**Table 1:** The antibacterial activity of different concentration at AgNPs (mm)

Concentration (mg/mL)	*E. coli* ATCC 11229**	*P. aeruginosa* ATCC 27853*	*S. aureus* ATCC 25923*	*S. epidermidis* ATCC 35984*	*S. mutans* ATCC 25175**
0.625	7.7 ± 0.2	11.3 ± 0.1	17.2 ± 0.2	14.3 ± 0.4	10.7 ± 0.2
1.25	7.9 ± 0.2	12.3 ± 0.2	17.4 ± 0.2	14.7 ± 0.3	11.1 ± 0.1
2.5	8.1 ± 0.3	12.6 ± 0.2	17.6 ± 0.2	14.9 ± 0.4	11.2 ± 0.2
5	8.2 ± 0.2	13.5 ± 0.2	18.2 ± 0.4	15.1 ± 0.2	11.4 ± 0.1
10	8.3 ± 0.1	14.2 ± 0.4	18.5 ± 0.3	15.4 ± 0.3	11.9 ± 0.2
20	8.8 ± 0.3	14.5 ± 0.1	18.6 ± 0.4	15.5 ± 0.3	12.3 ± 0.1
40	9.5 ± 0.4	14.9 ± 0.2	19.0 ± 0.4	15.7 ± 0.1	12.9 ± 0.4

The data are presented as the mean (± SD) of three replicates

^*^Correlation is significant at the 0.05 level (Pearson correlation)

^**^Correlation is significant at the 0.01 level (Pearson correlation)

### Anti-Biofilm

The anti-biofilm activity of AgNPs was evaluated against five pathogenic bacterial strains using a 96-well microtiter plate assay. Table 2 illustrates the anti-biofilm of AgNPs against *E. coli* ATCC 11229, *P. aeruginosa* ATCC 27853, *S. aureus* ATCC 25923, *S. mutans* ATCC 25175, and *S. epidermidis* ATCC 12228. AgNP inhibited *P. aeruginosa* and *E. coli* biofilm formation by 96.21 and 94.54% (40 mg/mL), respectively, while *S. epidermidis* was inhibited by 68.53% (0.156 mg/mL).

**Table 2:** Biofilm inhibition of different concentration at AgNPs (%)

Concentration (mg/mL)	*E. coli* ATCC 11229**	*P. aeruginosa* ATCC 27853**	*S. aureus* ATCC 25923*	*S. epidermidis* ATCC 35984**	*S. mutans* ATCC 25175*
0.156	91.49 ± 1.05	94.91 ± 0.63	71.49 ± 2.77	68.53 ± 1.03	77.33 ± 1.15
0.313	92.08 ± 0.65	95.10 ± 0.33	71.99 ± 0.50	68.86 ± 0.15	77.80 ± 0.24
0.625	92.59 ± 0.67	95.28 ± 0.01	73.76 ± 0.52	69.01 ± 0.87	78.45 ± 1.09
1.25	92.93 ± 0.01	95.46 ± 0.22	78.01 ± 0.55	69.77 ± 1.50	82.37 ± 0.89
2.5	93.16 ± 0.01	95.49 ± 0.39	83.62 ± 1.46	69.84 ± 0.01	84.82 ± 0.77
5	93.34 ± 0.08	95.66 ± 0.43	85.39 ± 0.99	70.48 ± 0.01	85.19 ± 0.75
10	93.63 ± 0.17	95.77 ± 0.36	85.82 ± 0.98	70.95 ± 0.15	85.33 ± 0.50
20	94.08 ± 0.34	96.07 ± 0.25	87.30 ± 1.24	71.90 ± 0.14	86.59 ± 0.47
40	94.54 ± 0.46	96.21 ± 0.01	88.44 ± 0.59	75.08 ± 0.01	88.17 ± 0.19
**Biofilm (OD)**	**2.62**	**3.9**	**1.41**	**0.63**	**1.35**
	** + + + **	** + + + **	** + + **	** + **	** + + **

The data are presented as the mean (± SD) of three replicates

^*^Correlation is significant at the 0.05 level (Pearson correlation)

^**^Correlation is significant at the 0.01 level (Pearson correlation)

### Antioxidant Activity

Antioxidant activity of AgNPS at different concentrations was investigated using four different methods (2,2-diphenyl-1-picrylhydrazyl (DPPH) free radical scavenging, iron chelating, hydroxyl radical scavenging, and superoxide anion scavenging activities) (Table 3). The highest DPPH activity of AgNPs was 80.93% at a concentration of 40 mg/mL, while the lowest activity was 34.41% at 0.156 mg/mL. In Fe^2+^ scavenging assays, AgNPs and ascorbic acid exhibited the highest antioxidant properties, with an antioxidant potential of 65.54% and 88.24%, respectively, at 40 mg/mL. The hydroxyl radical scavenging activity of AgNPs was found to be 90.17% at a concentration of 40 mg/mL. Table 3 shows for the superoxide anion radical scavenging activity of the AgNPs and AA. The results demonstrated that different concentrations of AgNPs (0.156–40 mg/mL) and AA showed a scavenging efficiency 66.01–72.49% and 80.37–95.15%, respectively.

**Table 3:** Antioxidant activities of different concentration at AgNPs and ascorbic acid (%)

Concentration (mg/mL)	DPPH free radical scavenging activity	AA	Iron chelating activity	AA	Hydroxyl radical removal activity	AA	Superoxide anion radical scavenging activity	AA
0.156^a^	34.41 ± 0.77^b,c,d,e,f,g,h,i^	75.24 ± 1.52	57.71 ± 0.67^g,h,i^	65.37 ± 2.7	73.45 ± 2.08	69.90 ± 3.14	66.01 ± 1.67	80.37 ± 3.70
0.313^b^	47.62 ± 0.36^a,c,d,e,f,g,h,i^	75.42 ± 1.99	58.02 ± 0.48^g,h,i^	68.14 ± 1.18	73.98 ± 2.37	72.12 ± 2.47	67.59 ± 2.36	80.74 ± 1.30
0.625^c^	55.42 ± 0.28^a,b,d,e,f,g,h,i^	89.54 ± 1.46	60.03 ± 0.61^h,i^	72.14 ± 1.41	74.60 ± 3.82	75.13 ± 1.75	68.12 ± 2.48	81.34 ± 2.14
1.25^d^	68.64 ± 0.86^a,b,c,e,f,g,h,i^	92.28 ± 1.04	60.75 ± 1.17^i^	79.24 ± 1.74	76.19 ± 2.56	81.51 ± 1.72	69.84 ± 2.69	83.24 ± 1.74
2.5^e^	73.26 ± 1.26^a,b,c,d,g,h,i^	96.71 ± 1.47	61.21 ± 0.96^i^	80.25 ± 1.74	76.39 ± 2.47	82.98 ± 2.04	70.50 ± 2.72	87.74 ± 2.70
5^f^	75.57 ± 1.37^a,b,c,d,h,i^	97.81 ± 2.51	62.61 ± 1.29^i^	83.12 ± 1.25	76.68 ± 2.44	84.97 ± 1.18	71.03 ± 2.79	89.17 ± 3.40
10^g^	77.45 ± 0.61^a,b,c,d,e,i^	98.02 ± 1.24	63.19 ± 1.34^a,b^	84.72 ± 2.78	77.68 ± 1.66	86.45 ± 1.93	71.56 ± 2.76	94.00 ± 1.80
20^h^	79.97 ± 0.45^a,b,c,d,e,f^	98.86 ± 2.98	64.20 ± 1.36^a,b,c^	88.00 ± 3.13	78.83 ± 1.17	89.19 ± 1.45	72.09 ± 2.63	95.00 ± 1.45
40^i^	80.93 ± 1.04^a,b,c,d,e,f,g^	100.00 ± 1.54	65.54 ± 1.87^a,b,c,d,e,f^	88.24 ± 1.95	79.22 ± 1.70	90.17 ± 2.58	72.49 ± 2.28	95.15 ± 2.47

The data are presented as the mean (± SD) of three replicates

Lower cases indicate between doses statistical difference, *p* < 0.05 (one-way ANOVA-Tukey)

### Cytotoxicity

**Fig. 6:**
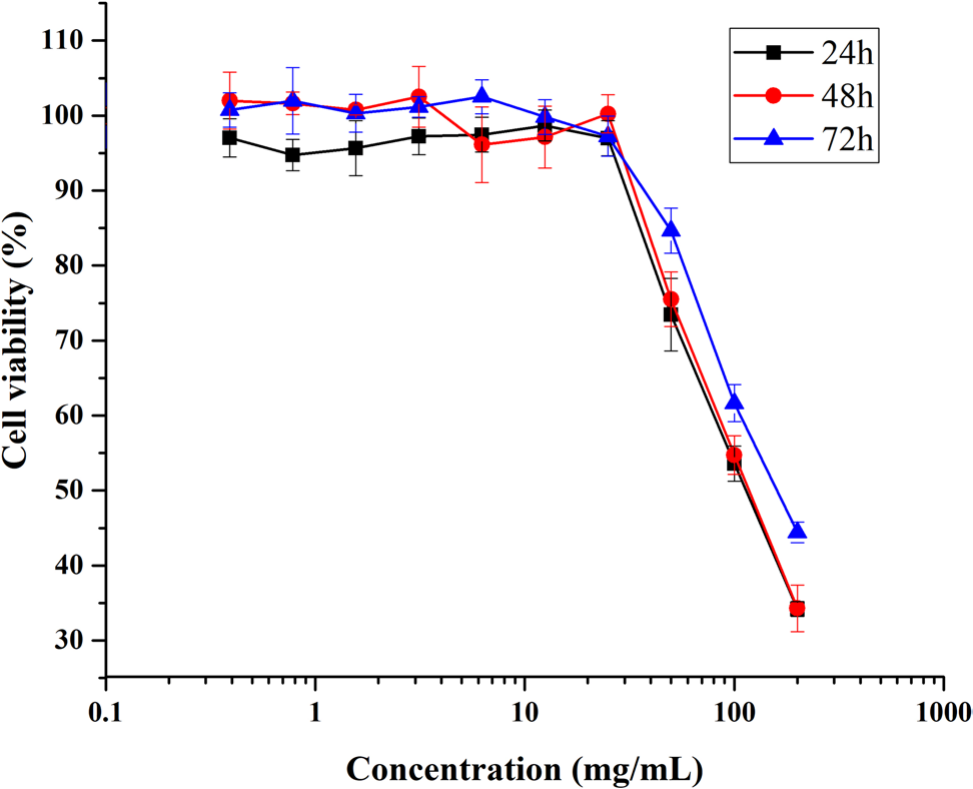
Cytotoxicity of AgNPs evaluated by MTT assay. Cell viability of L929 cell of AgNPs at 24, 48, and 72 h

L929 normal cell lines were used to assess the cytotoxic effects after 24, 48, and 72 h of incubation with different concentrations (0.39, 0.78, 1.56, 3.13, 6.25, 12.5, 25, 50, 100, and 200 µg/mL) of the synthesized nanoparticles. It is evident that low concentrations of AgNPs (0.39, 0.78, 1.56, 3.13, 6.25, 12.5, and 25 µg/mL) are non-toxic (Fig. 6). Additionally, after 24, 48, and 72 h of incubation, the IC_50_ values of AgNPs were 113, 117, and 162 µg/mL, respectively.

## Discussion

Many techniques have been discovered to synthesize AgNPs; however, due to sustainability, cost-effectiveness, and environmental concerns, there is an increasing interest in the green biological approach using plant and microbial sources. Although biological methods are regarded as safe, cost-effective, sustainable, and eco-friendly, culturing microorganisms could be time-consuming and presents challenges in accurately controlling the size distribution, shape, and crystallinity of the nanoparticles. Additionally, biological nanoparticles often need more monodispersity and faster production rates [2].

Extracellular synthesis is more commonly used because the reaction is simple, rapid, and the recovery and purification of the obtained nanoparticles (NPs) do not require additional steps as in intracellular synthesis [15, 29]. Biogenic synthesis has been favored in recent decades because it eliminates expensive chemicals and is environmentally friendly. Several bacterial species have been reported to synthesize AgNPs extracellularly, including *Lactobacillus* sp. [3, 8, 22, 30, 31], *Enterococcus* sp. [15], *Escherichia coli* [32], *Pseudomonas aeruginosa* [33], *Bacillus* species [34], *Solibacillus isronensis* [35], *Streptococcus pyogenes* [36], and *Oenococcus oeni* [37].

In this study, it was reported that a green, low-cost, and reproducible method for the biosynthesis of AgNPs using postbiotic of potential probiotics *L. salivarius* KC27L strain. Postbiotics function as reducing agents for silver ions, preventing nanoparticle aggregation, reducing toxicity, and enhancing their stability [8, 38]. The presence of AgNPs synthesized under optimum conditions (5 mM AgNO_3_, 6 h, 1:1 ratio, pH 3.5, and 30 °C) was confirmed by the presence of a UV–visible absorption peak at 435 nm. This peak, which typically falls between 400 and 500 nm, is a characteristic feature of AgNPs [39]. Additionally, the analysis of UV–visible spectra can provide insights into the shape and structure of biosynthesized AgNPs [40].

Optimizing physicochemical parameters such as contact time, AgNO_3_ concentration, pH, and temperature is crucial for enhancing the production rate of nanoparticles while improving their physical, morphological, and biochemical properties [41]. The incubation period was the initial factor taken into account for optimizing AgNPs biosynthesis [42]. After the synthesis of AgNPs for 6 h (Fig. 1a), the intensity of the absorbance decreased possibly because of the agglomeration of AgNPs at longer synthesis times [43]. Since the broadened peak indicates the formation of poly-disperse AgNPs [44], it was determined from the absorbance spectrum of AgNPs in our study that mono-disperse AgNPs were formed (Fig. 1b).

SEM and TEM could be used to analyze the surface morphology of nanoparticles, including their shape, size, size distribution, and aggregation patterns [45]. The researchers [24, 46] found the particle size of AgNP synthesized from lactic acid bacteria below 50 nm, similar to our study (Figs. 3 and 4). The size and uniformity of the green-synthesized AgNPs were measured using a nano zeta sizer, which employs dynamic light scattering (DLS) to determine the average particle size and polydispersity index (PDI) [37]. According to DLS analysis, the sizes of AgNPs samples were determined to be larger than the value determined by SEM and TEM. Due to the formation of a hydrodynamic layer around particles in aqueous solutions, the particle diameter measured by DLS can be overestimated compared to electron microscopy results [47]. The PDI reflects the degree of size variation in a sample. A PDI value above 0.7 signifies a polydispersity sample with a wide range of particle sizes, whereas a value below 0.7 indicates a monodisperse sample with relatively uniform particle sizes [48]. The samples with PDI values 0.309 can be characterized as the monodisperse form. The zeta potential reflects the surface charge and stability of nanoparticles. High positive or negative zeta potentials (above + 30 mV or below − 30 mV) contribute to stability by minimizing particle aggregation through electrostatic repulsion [49]. AgNPs had negative zeta potential (− 18.6 mV) because of the presence of a higher concentration of biomolecules. These findings suggest that stabilized with biomolecules carried negative charges and the NPs were stable due to the powerful repulsion between the NPs. El‑Hawary et al. [46] showed that synthesized AgNPs have a zeta potential of − 7.11 mV value. FTIR analysis can detect interactions between silver salts and extracellular proteins that play a role in AgNP synthesis. It has also been suggested that this method could reveal information about the binding of proteins to AgNPs, which may contribute to their stability [50]. In this synthesis using *L. salivarius* KC27L postbiotic, AgNPs exhibited characteristic peaks at 3167 cm^−1^ (3227 cm^−1^ in the postbiotic), 2922 cm^−1^ (2974 cm^−1^), 1637 cm^−1^, 1526 cm^−1^ (1581 cm^−1^), 1212 cm^−1^ (1236 cm^−1^), and 1052 cm^−1^, respectively (Fig. 5). These peaks were assigned to hydroxyl (− OH), aliphatic C-H stretching, amide I band (carbonyl stretching of the peptide bond), amide II band (N–H bending and C-N stretching), C-N stretching, and carbonyl stretching of the carboxyl group, respectively. The observed shifts in the peak positions indicate interactions between the functional groups during the formation of AgNPs [22]. The FTIR spectrum suggested that functional molecules, including biopolymers such as proteins, enzymes, and amino acids produced by probiotic bacteria, may play a role in both the synthesis and stabilization of AgNPs. The results obtained in the study are like the studies of Yusof et al. [22].

As a result of the literature searches, a study on silver nanoparticle synthesis using cell supernatant of the *Ligilactobacillus salivarius* MTCC-15009 strain was found [51]. They reported that AgNPs-LS were characterized using UV–vis spectroscopy, FTIR, TEM, DLS, and zeta potential analysis. UV–vis spectroscopy revealed a peak at 438 nm, confirming nanoparticle formation. TEM images showed spherical and polyhedral shapes, while FTIR identified functional groups (O–H, C = O, C-O–H, and C–C) involved in reduction and capping. DLS analysis determined a mean diameter of 79.47 nm with a polydispersity index of 0.60, and zeta potential analysis showed a negative surface charge of − 14.53 mV.

Synthesis of alternative antimicrobial compounds could potentially solve the widespread problem of antibiotic resistance. Therefore, the bactericidal properties of metal nanoparticles against pathogenic bacteria are important for their practical applications. It is particularly important that metal nanoparticles have anti-bacterial activity, which makes it difficult for bacteria to develop resistance [52]. In the study, the agar well diffusion method was employed to assess antimicrobial activity. The antimicrobial activities significantly increased as the concentration of AgNPs raised (*p* < 0.05/0.01) (Table 1). Our data is close to the results with prior investigations [9, 14, 53] demonstrating the antibacterial activity of green-synthesized AgNPs against pathogenic bacteria. The main mechanisms of the antibacterial activity of AgNPs have yet to be elucidated. Nevertheless, several hypotheses propose that AgNPs exert their antibacterial effects through various mechanisms, including (1) the production of reactive oxygen species, (2) damage to the cell wall and cytoplasmic membrane, (3) inactivation of ribosomes, and (4) the release of silver ions via oxidation [46, 54]. Researchers were reported that antimicrobial activity increased as the size of AgNPs decreased, and AgNPs with sizes smaller than 50 nm exhibited high antimicrobial properties [9, 14, 53]. Our findings are consistent with these results. Although there is one study on the antimicrobial activities of AgNPs synthesized from *L. salivarius* supernatant against *Chromobacterium violaceum*, *P. aeruginosa*, *S. aureus*, *E. coli*, and *Serratia marcescens* [51], our study is among the first to investigate the antimicrobial activities of AgNPs at different concentrations against *Streptococcus mutans* and *Staphylococcus epidermidis*.

Biofilm is a complex, three-dimensional structure formed by microbial cells adhering tightly to each other and a surface. These structures are commonly found in medical implants, industrial facilities, and natural environments. Biofilms resist antibiotics and disinfectants, leading to chronic infections and treatment difficulties [55, 56]. Biofilms are difficult to eliminate because they protect bacterial cells from harmful external conditions such as biocides, temperature, drying, and the host immune system [24]. New antimicrobial agents are needed to combat biofilm, which acts as a barrier to effective penetration and activity of antimicrobials. In this study, a dose-dependent biofilm inhibition increased in all bacteria (*p* < 0.05/0.01) (Table 2). The results were consistent with the results of Kanmani and Lim [12], who reported a dose-dependent increase in biofilm formation by *P. aeruginosa* and *E. coli*. Zeinivand et al. [57] investigated the anti-biofilm activity of AgNPs synthesized using exopolysaccharide from *L. paracasei* MN 809528 against six pathogenic microorganisms. Exposing these pathogens to AgNPs at concentrations ranging from 0.78 to 400 μg/mL showed that *E. coli* exhibited the highest biofilm inhibition (78.70%), followed by *P. aeruginosa* (72.80%). Among LAB, only one study was found to determine the anti-biofilm activity of AgNPs synthesized using postbiotic of *L. salivarius* cell-free supernatants against *S. marcescens*, *C. violaceum*, and *P. aeruginosa* [51]. Biofilm extracellular polymeric substances (EPS) form a complex, charged matrix that acts as a barrier to nanoparticles. NPs interact with biofilms in a three-stage process involving transfer, attachment, and migration [58]. The physicochemical properties of NPs, EPS, and the environment influence the efficiency of these stages. The NP size, EPS pore structure, and water channels govern NPs penetration into the biofilm. While these processes are influenced by particle charge and size, many aspects of NP-biofilm interactions still need to be better understood [59–61].

Reactive oxygen species generated from endogenous and exogenous sources contribute to various diseases when produced excessively. While biological systems possess endogenous antioxidant defenses, modern lifestyles and environmental factors often overwhelm these, leading to oxidative stress. This imbalance promotes the formation of harmful singlet and triplet oxygen atoms, causing cellular damage. To counteract this, developing exogenous antioxidants from natural sources is crucial for enhancing human health and combating chronic diseases [62].

The DPPH assay is a simple, rapid, and reliable method to assess the antioxidant capacity [62]. In this study, the DPPH radical scavenging activity was significantly different between doses of AgNPs (*p* < 0.05). Compared to the standard AA, AgNPs exhibited a lower scavenging ability (Table 3). These results are consistent with previous findings [9, 14]. Prema et al. [14] reported that the DPPH radical scavenging activity of AgNPs as 82.92% at a concentration of 120 mg/mL, while in our study, AgNPs showed 80.93% scavenging activity at 40 mg/mL. The DPPH scavenging test is based on the principle that antioxidants transfer electrons or hydrogen ion to neutralize the DPPH free radical, converting it into DPPH-H (hydrazine) [63]. Iron chelating activity is a property of a substance that binds iron ions (Fe^2+^ or Fe^3+^) to itself, preventing iron from interacting with other molecules [64]. The Fe^2+^ chelating activity of different concentrations AgNPs was rise linearly in a dose-dependent manner (*p* < 0.05). These results corroborate the findings of earlier research [64, 65]. Batir-Marin et al. [63] informed that that AgNPs synthesized using *Equisetum sylvaticum* L. exhibited 85% iron chelation activity at a concentration of 2.5 mg/mL. Our study is one of the first studies in which the iron chelating activity of AgNPs synthesized from lactobacilli-postbiotic is evaluated. The AgNPs exhibited strong iron-binding properties, suggesting their antioxidant activity was linked to their ability to chelate iron. The chelating activity of the AgNPs to compete with ferrozine for the ferrous ions could be estimated by the measurement of the color reduction [64]. The iron-sequestering capacity of AgNPs could be exploited in localized anti-infective strategies, as microorganisms require iron for survival against leukocyte-mediated immune responses [1, 63]. Hydroxyl radical (OH•) is a highly reactive and harmful-free radical. It can cause oxidative stress in our cells, leading to DNA damage, protein degradation, and lipid peroxidation [66]. The results showed that there was no significant difference between the doses. Synthesized AgNPs may have the potential to scavenge hydroxyl radicals, highly reactive species capable of damaging various biomacromolecules within living cells, making this research one of the initial studies of the hydroxyl radical scavenging properties of LAB-synthesized AgNPs. Our findings corroborate the earlier research by Batir-Marin et al. [63], which demonstrated that Es-AgNPs synthesized from *Equisetum sylvaticum* L. exhibited a potent hydroxyl radical scavenging capacity, achieving 90% scavenging at a concentration of 2.5 mg/mL. Superoxide anion (O_2_• −), which is closely associated with many inflammatory diseases, is a weak oxidant. However, it leads to the formation of strong and dangerous hydroxyl radicals and singlet oxygen, which contribute to oxidative stress [64]. The study showed that there was no significant difference between the doses (Table 3). Moteriya and Chanda [67] observed a superoxide radical scavenging activity of 85% for AgNPs synthesized using a *Caesalpinia pulcherrima* stem extract at a concentration of 180 μg/mL, whereas Mata et al. [68] reported a lower scavenging activity of 52% for *Abutilon indicum*-synthesized AgNPs at the same concentration.

The observed high antioxidant activity (at all methods) of biogenic AgNPs in this study could be attributed to their ability to eliminate singlet and triplet oxygen, decompose peroxides, or quench-free radicals [69]. Lactic acid bacteria possess a wide array of primer and secondary metabolites (lactic acid, H_2_O_2_, exopolysaccharide, bacteriocin, bacteriocin like substances, exopolysaccharides, vitamin, diacetyl, acetoin, etc.) that are highly useful as reducing and stabilizing agents [70]. While research on using NPs as antioxidant agents is ongoing, studies involving the synthesis of AgNPs from LAB-derived postbiotic remain limited. Therefore, the presence of postbiotic bioactive compounds in *L. salivarius* KC27L strain may contribute to the antioxidant properties of AgNPs. These AgNPs with antioxidant properties could be used as a potential strategy to reduce oxidative stress. AgNPs synthesized from *L. salivarius* KC27L postbiotic might be an alternative to synthetic antioxidants.

According to do MTT results, at the end of 72 h, it was determined that the sample at 6.25 µg/mL AgNPs concentration had the highest cell viability level with 102.51%. These data indicate that low concentrations (0.39–25 µg/mL) of AgNPs did not cause adverse effects on cell health (Fig. 6). There is one study on the cytotoxicity of AgNPs synthesized from lactic acid bacteria with normal cells (L929) was found. Viorica et al. [71] indicated that the viability of L929 cells decreased to 50% at concentrations above 200 μg/mL of bioactive silver composites synthesized from *Lactococcus lactis* 56 KY484989. Previous studies were evaluated the cytotoxicity of AgNPs using L929 normal cell lines [72, 73]. Składanowsk et al. [72] reported that AgNPs, synthesized from *Streptomyces* sp. NH28 strain, at concentrations of 1 and 5 µg/mL did not reduce the viability of L929 cells, suggesting no cytotoxic effects. Similarly, Mohanta et al. [73] found a gradual decrease in cell viability with increasing concentrations of AgNPs and observed that at a concentration of 1000 µg/mL AgNPs, L-929 cell viability was reduced to 74.38%. The cytotoxic effects of metal NPs are primarily attributed to the release of soluble metal ions within cells, which subsequently trigger the production of reactive oxygen species (ROS). This mechanism, involving both the cell’s proinflammatory response to metal NPs and their unique surface characteristics as a redox system, contributes to the harmful impacts on healthy cells [74]. In studies involving AgNPs, it has generally been reported that smaller AgNPs exhibit greater cytotoxic effects compared to larger nanoparticles [72, 73, 75]. However, the relationship between the size and toxicity of AgNPs in human cells has yet to be fully understood. The cytotoxicity of AgNPs synthesized from LAB has mostly been studied in cancer cells. This study is one of the first to evaluate the cytotoxicity of AgNPs synthesized from postbiotic of probiotic bacteria in normal cells. Thus, a more comprehensive evaluation of future use in biotechnological fields such as food, pharmaceuticals, and cosmetics will be facilitated, and their potential risks will be reduced.

## Conclusion

This study investigated a biogenic method to synthesize AgNPs by utilizing the postbiotic from the probiotic *Ligilactobacillus salivarius* KC27L. Microbial-based green synthesis used in the biosynthesis of nanoparticles is highly advantageous due to its ease of control and the ability to perform a large-scale synthesis [17]. In addition to these advantages, since microbial components act as reducing and stabilizing agents, NPs do not require any other reducing and stabilizing agents. The green synthesis of AgNPs using potential probiotic bacteria *L. salivarius* KC27L postbiotic offers a promising alternative to the toxic chemical methods currently employed, expanding their potential applications while minimizing environmental harm. These include their use as antimicrobial agents in healthcare settings, anti-biofilm coatings for medical devices and industrial surfaces, components in antioxidant therapies to combat oxidative stress–related conditions, and environmentally sustainable biomaterials for applications in water treatment, food preservation, and agriculture. By leveraging the bioactive properties of probiotic-derived postbiotics, this method enhances both the biocompatibility and functional versatility of AgNPs, making them suitable for a wide range of biotechnological and industrial applications. Although there are studies in which the antioxidant, antibacterial, anti-biofilm activities, and cytotoxicity of AgNPs synthesized from lactobacilli are determined, our study is one of the first studies in which the antioxidant, antibacterial, anti-biofilm activities, and cytotoxicity of AgNPs synthesized using *L. salivarius* postbiotic are evaluated together.

## Data Availability

No datasets were generated or analysed during the current study.
